# Evaluation of implant-materials as cell carriers for dental stem cells under *in vitro* conditions

**DOI:** 10.1186/s40729-014-0002-y

**Published:** 2015-02-12

**Authors:** Martin Gosau, Sandra Viale-Bouroncle, Hannah Eickhoff, Esthera Prateeptongkum, Anja Reck, W Götz, Christoph Klingelhöffer, Steffen Müller, Christian Morsczeck

**Affiliations:** 1 Department of Cranio- and Maxillofacial Surgery, Hospital of the University of Regensburg, Franz-Josef-Strauss-Allee 11, 93053 Regensburg, Germany; 2 Department of Oral and Maxillofacial Surgery, Paracelsus Medical University Nuernberg, Breslauer Str., 201, 90471 Nürnberg, Germany; 3 Department of Orthodontics, Oral Biology Laboratory, Dental Clinic, University of Bonn, Regina-Pacis-Weg 3, 53113 Bonn, Germany

**Keywords:** Dental stem cells, Hydroxyapatite, Allograft product, Osteogenic differentiation, Silicone

## Abstract

**Background:**

Dental stem cells in combination with implant materials may become an alternative to autologous bone transplants. For tissue engineering different types of soft and rigid implant materials are available, but little is known about the viability and the osteogenic differentiation of dental stem cells on these different types of materials. According to previous studies we proposed that rigid bone substitute materials are superior to soft materials for dental tissue engineering.

**Methods:**

We evaluated the proliferation, the induction of apoptosis and the osteogenic differentiation of dental stem/progenitor cells on a synthetic bone-like material and on an allograft product. The soft materials silicone and polyacrylamide (PA) were used for comparison. Precursor cells from the dental follicle (DFCs) and progenitor cells from the dental apical papilla of retained third molar tooth (dNC-PCs) were applied as dental stem cells in our study.

**Results:**

Both dental cell types attached and grew on rigid bone substitute materials, but they did not grow on soft materials. Moreover, rigid bone substitute materials only sustained the osteogenic differentiation of dental stem cells, although the allograft product induced apoptosis in both dental cell types. Remarkably, PA, silicone and the synthetic bone substitute material did not induce the apoptosis in dental cells.

**Conclusions:**

Our work supports the hypothesis that bone substitute materials are suitable for dental stem cell tissue engineering. Furthermore, we also suggest that the induction of apoptosis by bone substitute materials may not impair the proliferation and the differentiation of dental stem cells.

**Electronic supplementary material:**

The online version of this article (doi:10.1186/s40729-014-0002-y) contains supplementary material, which is available to authorized users.

## Background

While bone substitute materials are routinely used, especially vertical bone, augmentation of the jaws is still a problematic step. Dental stem cells in combination with bone substitute materials may accelerate the augmentation of alveolar bone and perhaps, stem cell-based therapies can become an alternative to autologous, allogenic, or synthetic bone transplants and substitutes [1,2]. However, scaffolds are required for cell delivery, and here, commercially available bone substitute materials could be an excellent source for dental tissue engineering.

For more than 10 years, human dental stem cell research has focused on the identification and characterization of human stem/progenitor cell populations, which can be isolated, for example, from retained third molars of juvenile patients [3]. One example for such type of dental stem cells are undifferentiated cells from the dental follicle (DFCs) [4,5]. These highly proliferative cells can be differentiated *in vitro* into periodontal ligament (PDL) cells, cementoblasts and osteoblasts, and into PDL-like cells *in vivo* [4]. Preliminary results from animal studies suggested that DFCs have also a good osteogenic differentiation potential and could be an excellent source for the regeneration of craniofacial bone [6]. Another excellent source for cellular therapies of mineralized tissues is progenitor cells from the dental apical papilla of retained third molar tooth (dNC-PCs) [7]. These dental cells differentiate into osteoblast-like cells after the induction with osteogenic differentiation medium under *in vitro* conditions and under *in vivo* conditions in immunocompromised mice [8].

For the osteogenic differentiation under *in vivo* conditions, stem cells are combined actually with hydroxyl-apatite (HAP) or β tricalcium phosphate (TCP) scaffolds [4,9]. Although this is routinely applied, we know only little about the adherence and the viability of dental progenitor cells on these implant materials. Conversely, an optimal bone substitute material has not been identified so far for different dental stem cell types. In a recent study, we investigated, therefore, cell survival/proliferation and cell differentiation of DFCs in combination with a commercially available TCP [10]. Here, DFCs attached on TCP and cell numbers increased after 6 days of cultivation. We showed that DFCs had a typical flattened-shaped morphology with close contacts to the bone substitute material [10]. Interestingly, the gene expression of osteogenic markers such as osteopontin or RUNX2 was increased, and the alkaline phosphatase (ALP) activity was induced on TCP in differentiated DFCs [10]. All these data support the assumption that TCP could be the optimal scaffold for a successful differentiation protocol of DFC.

Unfortunately, an additional study showed that TCP induced apoptosis in DFCs [11]. However, the induction of apoptosis exposed a risk for cellular therapies. We decided therefore to evaluate additional implant materials for the identification of a suitable scaffold for dental stem cells. Soft materials such as silicone are successfully used in regenerative medicine, and they are suitable for tissue engineering, but, however, we propose that rigid and bone-like materials are superior for dental tissue engineering than soft implant materials. Therefore, this study evaluated and compared solid bone substitute materials with elastic materials such as silicone or polyacrylamide (PA). This study investigated the proliferation, the induction of apoptosis, and the osteogenic differentiation of DFCs and dNC-PCs after the attachment on implant-materials.

## Methods

### Cell culture

The isolation and characterization of DFCs and dNC-PCs were described in previous studies [4,7,12]. DFCs were routinely cultivated in DMEM (Sigma-Aldrich, St. Louis, MO, USA) supplemented with 10% fetal bovine serum (Sigma-Aldrich, St. Louis, MO, USA) and 100 μg/ml penicillin/streptomycin (standard cell culture medium). dNC-PCs were cultivated in DMEM (Sigma-Aldrich) supplemented with 15% fetal bovine serum (Sigma-Aldrich) and 100 μg/ml penicillin/streptomycin (standard cell culture medium). For experiments, both cell types were used after passage 6. DFCs and dNC-PCs expressed typical markers for dental stem cells such as CD105, Nestin, and STRO-1 (Additional file 1: Figure S1).

### Preparation of polyacrylamide materials

Five milliliter of PA gel solution with the concentration of 8% acrylamide and 0.06% bis-acrylamide (Bio-Rad, Hercules, CA, USA) were mixed and degas under vacuum for at least 20 min to remove oxygen. Then, 30 μl of 0.1 mg/mL ammonium persulfate (Sigma-Aldrich, St. Louis, MO, USA) and 20 μl TEMED (Applichem, Omaha, NE, USA) were added and placed into the mini protean casting strand and frame (Bio-Rad) to form 1-mm thickness of substrate. After letting the gel to polymerize for 30 to 45 min, gently remove and rinse gel with 50-mM HEPES, pH 8.5 (Applichem, Omaha, NE, USA). PA gel was then cut into circular shape with 14 mm diameters and placed in 24 well plates for the experiment. Sulfo-SANPAH (Pierce Biotechnologies, Rockford, IL USA) 0.5 mg/mL in 50-mM HEPES, pH 8.5 was pipetted onto the surface and exposed to the UV light for photoactivation procedure. After photoactivation, the substrate was washed several times in 50-mM HEPES. A 0.2 mg/mL of type I collagen (Sigma-Aldrich, St. Louis, MO, USA) was then layered onto the surface of gel and incubated 4 h at room temperature or overnight at 4°C on a shaker. After washing with PBS, the gels were stored in PBS at 4°C. Before platting the cells, the gel was exposed to UV for 15 min for the sterilization and replace PBS with complete culture medium for 1 h at 37°C.

### Implant materials

The bone substitutes Maxgraft® (AP) and Maxresorb® (SB) were obtained from the company Botiss (botiss dental GmbH, Berlin, Germany). Maxgraft® is a sterile, high-safety allograft product (AP), derived from human donor bone. It is processed by an audited and certified bone bank (Cells^+^ Tissue Bank Austria, Berlin, Germany). In contrast, Maxresorb® is a fully synthetic bone graft substitute (SB) with controlled resorption properties. It is a homogenous composition of 60% hydroxyapatite and 40% beta-tri-calcium phosphate. SB maintains the volume and mechanical stability over a long time period. The osteoconductivity of SB is achieved by a matrix of interconnecting pores and a very high porosity of approximately 80%, as well as pore sizes from 200 to 800 μm (www.botiss.com). Experiments with AP and SB were done with solid blocks (10 × 10 × 20 mm cancellous block). PA was produced in our lab (see above), and silicone-based implant materials were obtained from Vivomed (Downpatrick, UK) as tubes. Silicone tubes were cut in pieces with a size which is similar to that of AP and SB.

Implant materials were washed with PBS or cell culture medium before use. DFCs and dNC-PCs were seeded onto materials for indicated periods of time. For the isolation of total RNA and the estimation of vital cell numbers, implant materials with cells were transferred to a fresh well with cell culture medium.

For the evaluation of apoptosis induction, cell culture eluates were produced by incubating 0.1 mL of bone substitutes or soft materials in 1-mL standard medium at 37°C for 24 h. This incubation step with the implant material was repeated twice with fresh cell culture media. Three eluates were pooled for cell culture experiments. DFCs were seeded onto cell culture plates and cultivated in standard cell culture media. After cell seeding (12 to 24 h), cell culture media were changed, and cells were cultivated in cell culture media with material eluates. After 24 h of cultivation, cells were harvested for flow cytometry analyses or protein isolation for Western blots (see below).

### Cell counting kit 8 assay

Numbers of vital cells were evaluated after days 1, 2, 3, and 6. For cell counting, cell cultures were incubated with the cell counting kit 8 (CCK8) ready to use solution according to manufactures instructions (Dojindo, Rockville, MD, USA). The optical density (O.D.) was measured at a wavelength of 450 nm. For the evaluation of the cell adherence (normalized to standard cell culture dishes), cell proliferation (normalized to cell number at day 1 of cell culture) relative cell numbers were calculated.

### Flow cytometry analysis

The induction of apoptosis in DFCs and dNC-PCs was evaluated by measuring the Cell Event® Caspase3/7 Green Flow cytometry assay (Life Technologies, Carlsbad, CA, USA). For the Caspase3/7assay, cells were cultivated in eluates as described above. After 24 h, cells were harvested by trypsin-EDTA treatment, washed with PBS, and stained first with Caspase3/7 Green Detection Reagent (25 min, 37°C). After this step 1-mM SYTOX® AADvanced dead cell stain solution was added to the sample (5 min, 37°C). Cell fluorescence was analyzed at 488-nm excitation and applied to standard fluorescence compensation. Emission of fluorescence was measured with 530/30 BP (Caspase3/7 Green Detection Reagent) and with 690/50 BP (SYTOX® AADvanced dead cell stain) filters. Cells positive for Caspase3/7 Green Detection Reagent were identified as apoptotic cells, while dead cells were positive for SYTOX® AADvanced dead cell stain. However, vital cells were negatively stained for both staining solutions.

### Western blotting

For protein isolation, cells were treated with lysis buffer (250 μl phosphatase, 100 mM Na3VO4, 137 mM NaCl, 200 mM Tris, 480 mM NaF, 1% NP-40, 10% Glycerol) on ice for 2 min. A protease-inhibitor (1 Protease Inhibitor Cocktail tablet from Roche) was included to minimize protein degradation. Cell lysates were placed on ice for 10 min. Protein samples were separated by SDS-polyacrylamide gel electrophoresis in pre-casted 12% Tris-glycine gels (Invitrogen, Waltham, MA, USA) and blotted to nitrocellulose membranes. Membranes were blocked with skimmed milk for 1 h and incubated with primary antibodies that were specific for proteins BAX (pro apoptotic protein), BCL2 (anti apoptotic protein), and β-Actin (housekeeper protein). Washed membranes were then incubated with a horseradish peroxidase-labeled secondary antibody. The detection of the secondary antibody was performed via chemiluminescence and X-ray films (GE Healthcare, Pewaukee, WI, USA).

### Osteogenic differentiation

DFCs were cultivated until sub-confluence (>80%) in standard cell culture medium before the differentiation starts with the osteogenic differentiation medium (ODM) comprised DMEM (PAA) supplemented with 10% fetal bovine serum (Sigma-Aldrich), 100 μmol/L ascorbic acid 2-phosphate, 10 mmol/L KH_2_PO_4_, 1 × 10^−8^ mol/L dexamethasone sodium phosphate (Sigma-Aldrich, St. Louis, MO, USA), HEPES (20 mmol/L) and 100 μg/ml penicillin/streptomycin. The differentiation was evaluated by qRT-PCR and ALP activity detection.

### ALP activity detection

Cells were washed with PBS buffer and lysed by shock freezing (−80°C). Diluted 1:1 in 1 × PBS, 100 mM p-nitrophenyl phosphate (Sigma) was added to each sample. After incubation at 37°C for 60 min, the reaction was stopped by adding 300 μL of 0.3 M NaOH and the liberated p-nitrophenol was measured at 405 nm. ALP activity values were normalized to total DNA concentration, which were determined by the Quant-iT PicoGreen dsDNA Assay (Invitrogen).

### Prime PCR arrays

For the evaluation of osteogenic marker expression, the Biorad PrimePCR array (Development - Hedgehog and PTH signaling pathways in bone and cartilage development) was used, which consists of the most important markers for the osteogenic differentiation. Total RNAs, which were isolated from differentiated dental cells at day 7, were reverse-transcripted with the iScript™ Advanced cDNA Synthesis Kit for RT-qPCR (Biorad) according to the manufacturers protocol. PCRs were made with SsoAdvanced™ Universal SYBR® Green Supermix (Biorad) on the StepOne real-time PCR machine (Life Technologies, Carlsbad, CA, USA). Results were analyzed with the PrimePCR™ Analysis Software (Biorad), and the output is presented as Clustergrams. While red tiles signify a high gene expression, black/gray and green tiles show a middle gene expression and a low gene expression, respectively. Black tiles with a cross designate no gene expression.

### Histology

Combinations of SB with dNC-PCs and AP with dental cells yielded from cell cultures after 7 days of osteogenic differentiation were fixed in 4% formaldehyde/0.1 M PBS at 4°C for at least 24 h. Tissues were decalcified with EDTA and subsequently dehydrated in an ascending series of ethanol and embedded in paraffin. Serial sections of 5 μm were cut in different planes for orientation and stained with hematoxylin-eosin (HE).

## Results

### Cell viability

**Figure 1:**
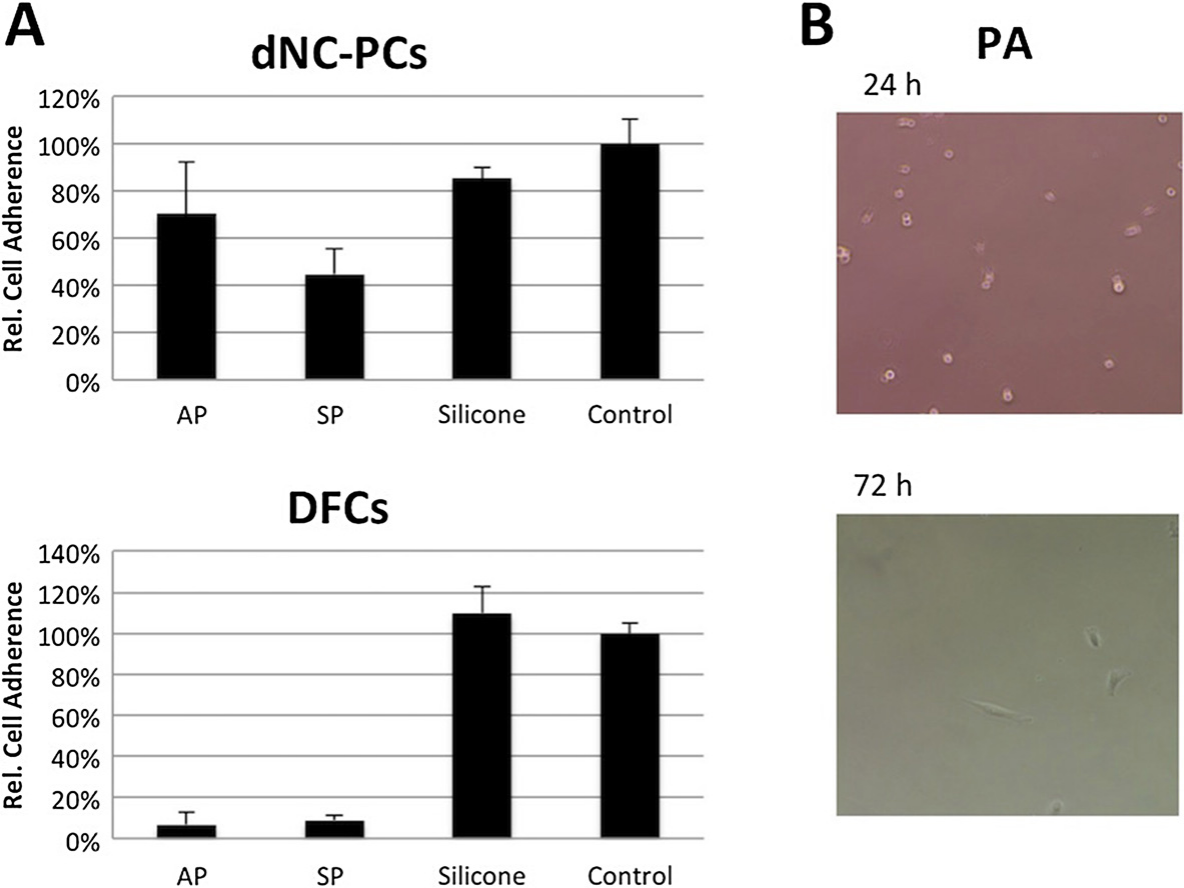
**Cell attachment on tested materials. (A)** Relative cell adherence of DFCs and dNC-PCs; **(B)** dental cells did little adhere on PA; representative pictures of DFCs.

**Figure 2:**
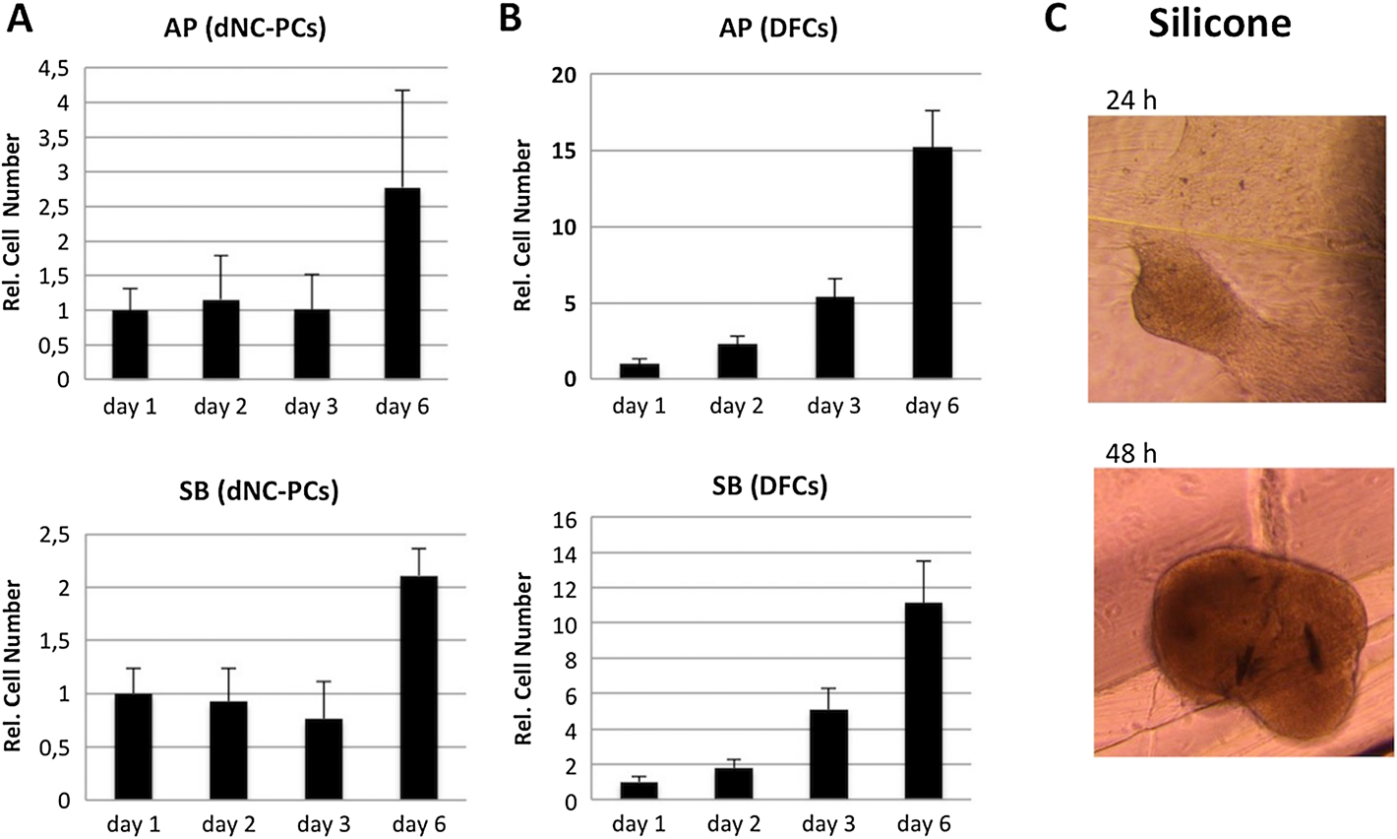
**C** **ell proliferation of dNC-PCs and DFCs on tested materials. (A)** and **(B)** Relative cell numbers;** (C)** spheroid cell clusters on silicone (representative pictures for DFCs); Silicone (24 and 48 h).

Dental cells were cultivated in standard cell culture media until passage 6. Cell adherence and cell proliferation/growth were measured for the estimation of cell viability on tested rigid and soft materials. In Figure 1, the cell adherence of dNC-PCs on bone substitute materials was better than that of DFCs. However, both dental cells types adhered very well on silicone. Unluckily, dental cells did not adhere on PA; only single cells survived for longer than 48 h (Figure 1B). For the evaluation of cell proliferation, relative cell numbers on implant materials were measured (Figure 2). While cell proliferation of dNC-PCs was moderate (Figure 2A), relative cell numbers of DFCs increased dramatically on bone substitute materials (Figure 2B). However, these results proved the viability of dNC-PCs and DFCs on SB and AP. Interestingly, dental cells formed large spheroid cell clusters on silicone, but cells lost their adherence to this material (Figure 2C), so numbers of silicone adherent cells decreased until day 6 of cell culture (data not shown).

**Figure 3:**
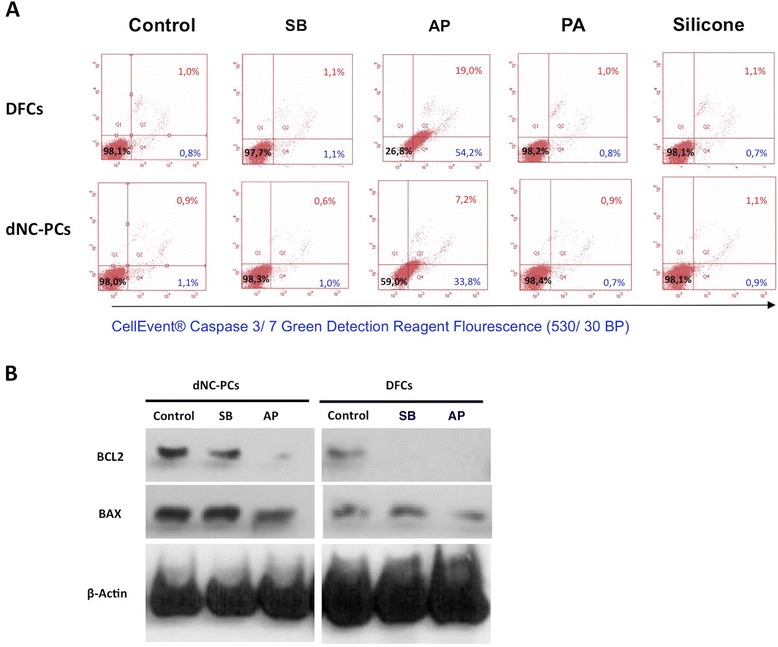
**Evaluation of programmed cell death (apoptosis) in dental stem cells. (A)** Flow cytometry analyses (for details materials and methods) show percentage of vital cells (black number), apoptotic cells (blue number), and dead cells (red number). (**B)** Western blot analyses show the expression of the pro-apoptotic marker BAX and the anti-apoptotic marker BCL2.

The induction of apoptosis and/or cell death was estimated by flow cytometry and western-blot analyses (Figure 3). While eluates of SB, PA, and silicone did neither induce cell death nor apoptosis, AP induced both cell death and apoptosis in DFCs and dNC-PCs. Both dental cell types expressed the pro-apoptotic marker BAX and the anti-apoptosis marker BCL2 under standard cell culture conditions (Figure 3B). However, BCL2 was not expressed on AP. Interestingly, BCL2 was also not expressed in DFCs after cultivation on SB. The low expression of BCL2 in DFCs may explain the low cell adherence on SB and AP (Figure 1).

### Osteogenic differentiation

**Figure 4:**
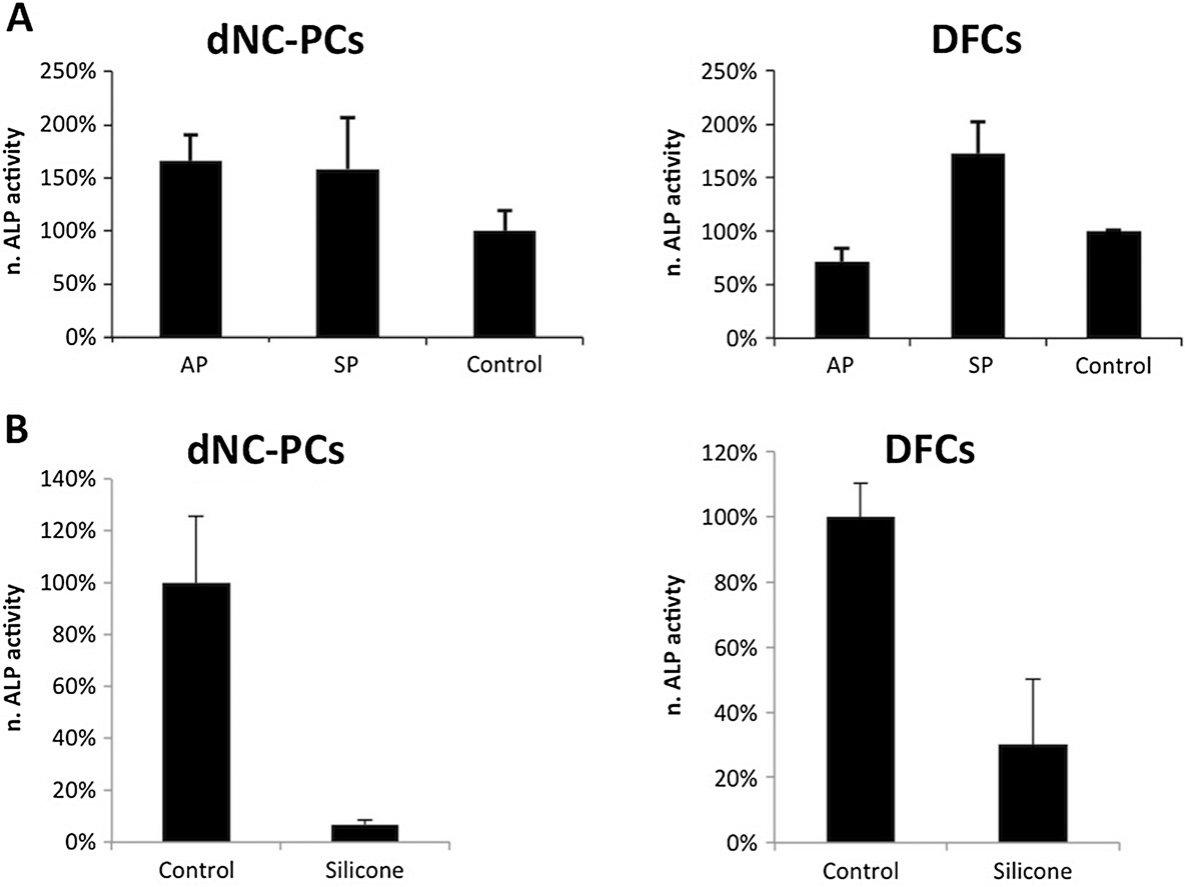
**Osteogenic differentiation of dental stem cells.** Normalized ALP activity of dNC-PCs and DFCs on AP and SB **(A)** and on silicone **(B)**. Cells were differentiated on standard cell culture dishes for control.

**Figure 5:**
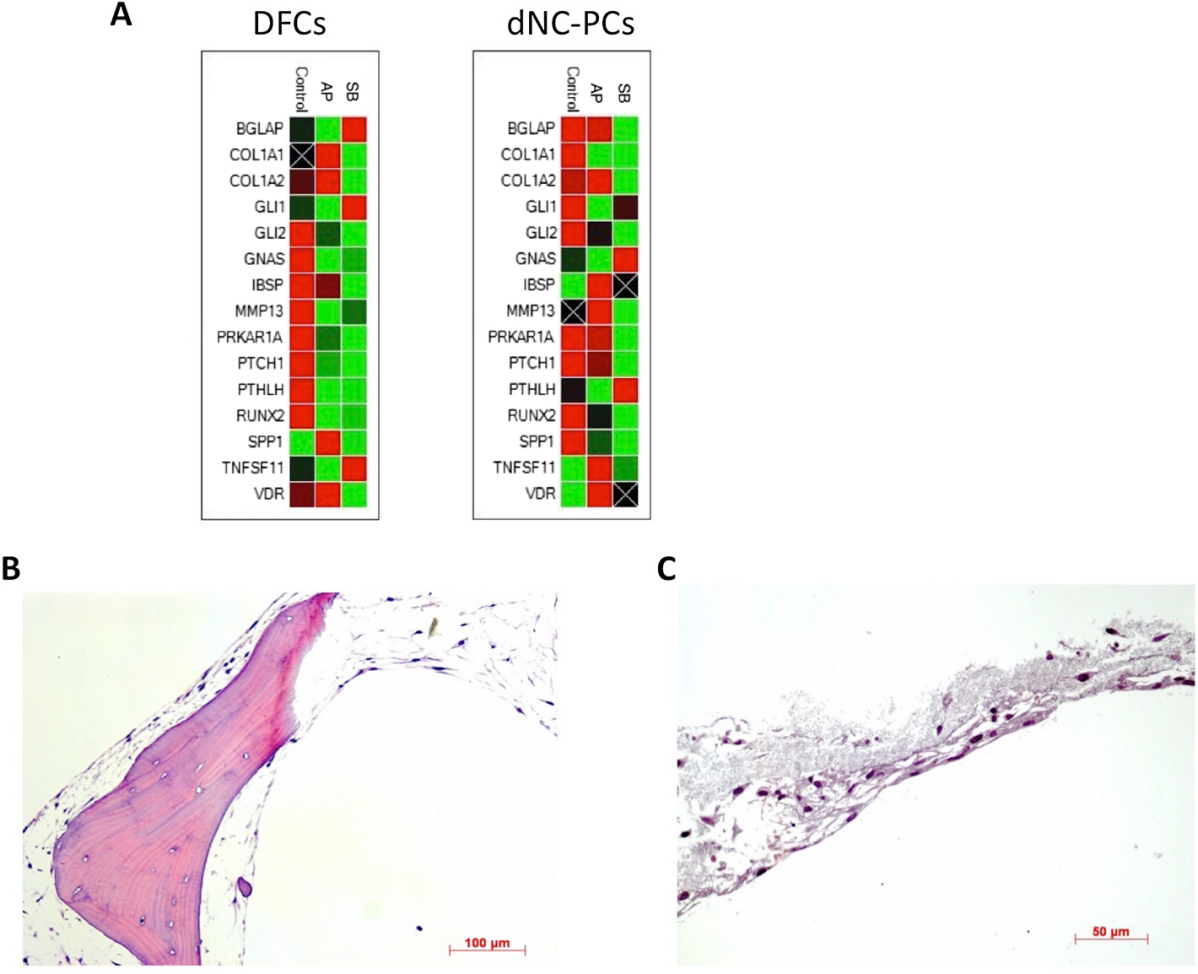
**Evaluation of osteogenic differentiation. (A)** Clustergram of PCR-array results; **(B-C)** histology of differentiated dental cells on AP **(B)** and SB **(C)**. Representative results are shown for dNC-PCs.

We measured the normalized ALP activity in dNC-PCs and DFCs after cultivation on tested materials (Figure 4). While ALP activities in dental cells on bone substitutes were increased or comparable to that of differentiated cells in standard cell culture dishes (control), the specific ALP activity was decreased on silicone (Figure 4B). A PCR array analysis showed that AP induced the expression of osteogenic differentiation markers (Figure 5A). Moreover, differentiated cells formed thick connective tissue like matrices on bone substitute materials (Figure 5B). These results reminded on the differentiation of osteogenic progenitor cells.

### PA after collagen I modification

**Figure 6:**
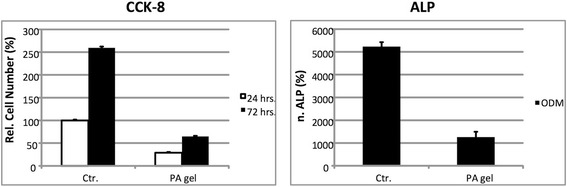
**Cultivation and osteogenic differentiation of DFCs on PA after modification with collagen I.** (Left) Relative cell number and (Right) normalized ALP activity.

The soft material PA was also treated with the extracellular matrix protein collagen to improve cell adherence. We tested representatively DFCs with collagen I modified PA. DFCs adhered and proliferated on modified PA, but, however, the specific ALP activity was reduced in comparison to that of DFCs on standard cell culture dishes (Figure 6). This reduction of the specific ALP activity was similar to that of silicone.

## Discussion

Scaffolds play an important role in tissue engineering. However, little is known about the proliferation and differentiation of DFCs and dNC-PCs on different types of materials. As we have learned from previous studies mechanical properties such as surface stiffness are decisive for a successful osteogenic differentiation of dental stem cells [13,14]. Moreover, we showed that bone substitute materials such as β-tricalcium phosphate (TCP) supports the osteogenic differentiation [10]. Our study proposed therefore that bone-like materials such as commercially available bone substitutes are superior for dental tissue engineering. Therefore, bone substitute materials SB and AP were compared with soft or connective tissue like materials. SB is synthetic and consists of 60% HAP and 40% TCP. In contrast, AP is an allograft product, which was derived from human donor bone. For comparison, two different soft materials silicone or PA were used in our study. Whereas silicone is routinely applied in regenerative medicine, the self-made PA scaffold has been very often used in cell biology studies [15].

dNC-PCs and DFCs attached on SB, AP, and silicone, but not on PA unless it was untreated. A modification with the extracellular matrix protein collagen permitted the attachment of dental cells. Interestingly, cell proliferation on silicone was hampered, because dental cells grew in non-attached spheroid cell clusters. This formation of spheroid cell clusters reminds on the neurogenic differentiation of DFCs [16-18]. The proliferation of DFCs on SB and AP was better than that of dNC-PCs, because the attachment of DFCs on these materials was lower than that of dNC-PCs. However, we conclude that bone substitute materials are suitable for dental cell attachment and proliferation. Our results for bone substitute materials are comparable to that of previous studies with different dental cell types. Kasaj and co-workers showed that cell adherence and cell proliferation of PDL cells on nanostructured HAP bone replacement grafts *in vitro* [19]. In another study, PDL cells adhere and proliferate on chitosan or on a combination of chitosan and nanostructured HAP [20]. In this setting, the combination of chitosan and nanostructured HAP was even favored by PDL cells. The adhesion and proliferation of dental pulp derived cells on HAP was demonstrated by Abe et al. [21].

In a previous study, we showed that TCP induced the programmed cell death (apoptosis) in DFCs [11]. Our new study investigated therefore the induction of apoptosis in dental cells. While SB and soft materials did not induce apoptosis or cell death, AP induced obviously cell death and apoptosis in dental cells. Here, the results for dNC-PCs and DFCs were almost the same. Interestingly, neither silicone nor PA induced the apoptosis in dental cells but did not also sustain the osteogenic differentiation of dental cells. Here, the ALP activity was strongly inhibited. Although no explanation for the induction of apoptosis by AP is available, the induction of apoptosis by AP does not correlate with the induction of the osteogenic differentiation. Both bone substitute materials sustained the differentiation, but only AP induced the expression of typical osteogenic differentiation markers. The induction of both osteogenic markers and apoptosis is very similar to that of our previous studies with TCP [10,11]. Interestingly, a study with pre-differentiated human cord blood stem cells showed also very similar effects on TCP [22]. They discovered a reduced number of pre-differentiated stem cells after long term cultures on TCP [22]. But although cell numbers decreased between days 1 and 7, the gene expression of osteogenic cell differentiation markers was increased [22]. In contrast, Marino et al. demonstrated that TCP scaffolds promoted both cell proliferation and osteogenic differentiation of human adipose stem cells [23]. However, additional studies are required to disclose the molecular relationship between apoptosis and the osteogenic differentiation.

Finally, we could show that surface modifications are important for the attachment and cell proliferation of dental cells (Figure 6). Our results are in accordance to the results obtained in previous studies. For example, modifications such as fibronectin coating of TCP or composites with a combination of polymer of poly glycolic-lactic acid (PGLA) with TCP may also influence cell attachment and proliferation of seeded cells [24,25]. Moreover, Seebach et al. showed that TCP products from different suppliers differ substantially in their morphology and that surface or porous structure seems to be of importance for the cell seeding and proliferation [25]. Unfortunately, a modification of PA with collagen did not improve the osteogenic differentiation of dental stem cells.

## Conclusions

In conclusion, our work supports our hypothesis that soft implant materials are not suitable for dental tissue engineering. Moreover, our study also supports the results of our previous studies with DFCs and TCP that induction of apoptosis did not impair the proliferation and the differentiation in dental stem cells.

## Additional file

Additional file 1:
**DFCs and dNC-PCs expressed typical markers for dental stem cells.**
