# Supplementary figures and images for: Evaluation of implant-materials as cell carriers for dental stem cells under in vitro conditions
Source: Int J Implant Dent. 2015 Feb 12;1(1):2. doi: 10.1186/s40729-014-0002-y (PMC5004001; doi:10.1186/s40729-014-0002-y)

## Slide 1
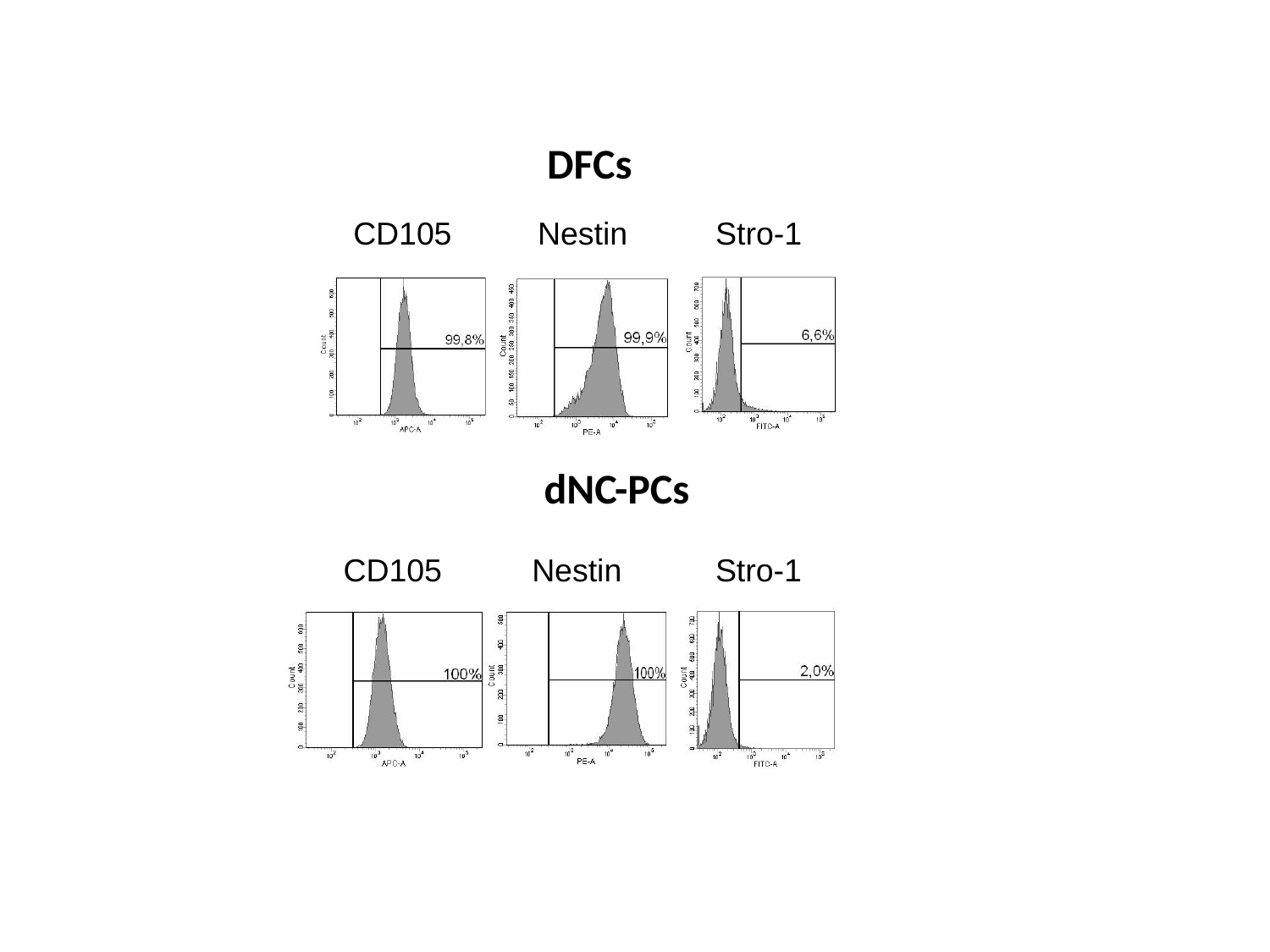

DFCs
CD105
Nestin
Stro-1
dNC-PCs
CD105
Nestin
Stro-1

Supplement: Additional file 1: — DFCs and dNC-PCs expressed typical markers for dental stem cells. [file 40729_2014_2_MOESM1_ESM.pptx]
